# No Role of Herpes Simplex Virus Type 2 (HSV-2) Infection on HIV Progression in Naïve HIV Patients

**DOI:** 10.22034/ibj.22.2.123

**Published:** 2018-03

**Authors:** Minoo Mohraz, Arezoo Aghakhani, Saeedeh Moayedi-Nia, Mohammad Banifazl, Alireza Janbakhsh, Setareh Mamishi, Afsaneh Karami, Anahita Bavand, Pegah Mirzapour, Amitis Ramezani

**Affiliations:** 1 Iranian Research Center for HIV/AIDS, Iranian Institute for Reduction of High-Risk Behaviors, Tehran University of Medical Sciences, Tehran, Iran; 2 Department of Clinical Research, Pasteur Institute of Iran, Tehran, Iran; 3 Iranian Society for Support of Patients with Infectious Disease, Tehran, Iran; 4 Kermanshah University of Medical Sciences, Kermanshah, Iran; 5 Pediatric Infectious Disease Research Center, Tehran University of Medical Sciences, Tehran, Iran; 6 Zanjan University of Medical Sciences, Zanjan, Iran

**Keywords:** Human immunodeficiency virus (HIV), Herpes simplex virus type 2 (HSV-2), Serology, CD4 lymphocyte count, Viral load

## Abstract

**Background:**

Herpes simplex virus type 2 (HSV-2) is a common infection in human immunodeficiency virus (HIV) patients and may accelerate HIV progression by rising HIV viral load and decreasing CD4 count. However, the available data regarding the influence of HSV-2 seropositivity on HIV progression in HIV individuals are inconclusive. Therefore, we aimed to determine HSV-2 seroprevalence in naïve HIV patients and normal controls and also investigate the relation of HIV viral load and CD4 count with HSV-2 seropositivity. Subsequently, we investigated the association of HSV-2 serostatus with changing in CD4 count and HIV viral load in our subjects, after one year follow-up.

**Methods:**

In this study, 116 naïve HIV patients and 85 healthy controls from Tehran, Iran were enrolled. HSV-2 IgG antibody was detected by ELISA. CD4 count was determined by flowcytometry, and serum HIV RNA copy numbers were determined using real-time PCR.

**Results:**

The prevalence of HSV-2 IgG was 18.1% in naïve HIV patients and 0% in the control group (*P* = 0.000). HSV-2 seroconversion was observed in 2.43% of HIV patients after one year. There was no significant difference regarding HSV-2 serostatus with CD4 count and HIV RNA viral load in our study cohort at baseline and after one year.

**Conclusion:**

Our results revealed that the prevalence and incidence of HSV-2 infection are low in our HIV cases, and it is negligible in the control group. However, it seems that HIV/HSV2 co-infection has no role on HIV infection acceleration.

## INTRODUCTION

Herpes simplex virus type 2 (HSV-2) is a common sexually transmitted infection[1,2] and the major etiology of genital ulcer disease throughout the world[3]. Moreover, it is a significant cofactor for acquiring human immunodeficiency virus (HIV) infection and can facilitate the transmission risk of HIV by twofold to threefold[4]. HSV-2 lesions act as HIV entry portals, and HSV-2 can enter into susceptible HIV target cells (such as CD4 cells), inducing the proliferation and activation of macrophages and T lymphocytes and leading to more susceptibility of these cells to HIV infection[5,6].

HSV-2 seroprevalence in HIV individuals is 60-90%[7], and it is three times higher in HIV patients than normal population[8]. HSV-2 can cause an incurable life-long viral infection and recurrent genital ulcers[9]. Clinical manifestations of HSV-2 vary from mild genital symptoms, in most HIV cases, to severe genital ulcers, in subjects with AIDS disease[10]. Investigations have shown that the frequency of subclinical shedding in HIV patients is higher than in uninfected HIV individuals[3,11]. HSV-2 can up-regulate HIV replication and increase HIV viral load, which is a key factor for HIV progression and transmission[12,13]. Moreover, HSV-2 reactivation is more common in HIV infection and results in elevation of HIV viral load in plasma and genital system[14,15]. Some surveys have investigated the influence of HSV-2 on HIV infection progression such as changing in HIV plasma viral loads or CD4 count, but their results were contradictory. It has also been indicated that HSV-2 seropositivity is associated with increasing HIV viral load in cases with early HIV infection[16], and individuals with prevalent HIV infection co-infected with HSV-2 have higher viral loads than subjects without HSV-2 infection[17]. These findings suggest a conception that HSV-2 infection may change HIV viral load and accelerate disease progression following initial HIV infection[18]. Accordingly, HSV suppressive therapy can decrease the HIV disease progression[19,20]. Some other studies found no association between HSV-2 serostatus and HIV viral load[18,21,22]. Therefore, data regarding the influence of HSV-2 seropositivity on HIV viral load in HIV patients are inconclusive. Besides, investigations in the effect of HSV-2 seropositivity on CD4 count have shown unconvincing results[18,21].

The primary aim of this study was to evaluate HSV-2 seroprevalence in normal controls and HIV patients not receiving highly active antiretroviral therapy (HAART). Our secondary goals were to compare the baseline HIV viral load and CD4 count among cases with and without HSV-2 co-infection and to investigate the association of HSV-2 serostatus with CD4 count and HIV viral load changes in naïve HIV subjects, after one year follow-up.

## MATERIALS AND METHODS

### Study population

**Fig. 1:**
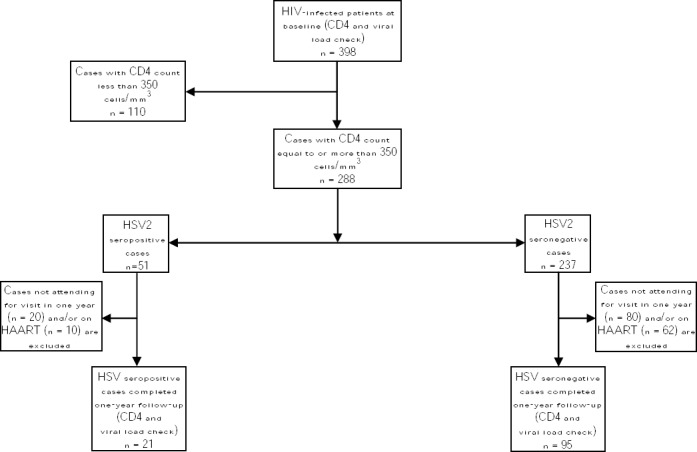
Flowchart of study participants and studying steps

In this study, 116 naïve HIV patients, who were referred to Iranian Research Center for HIV/AIDS in Tehran, Iran, and 85 healthy controls were consecutively enrolled in the study and followed-up from April 2014 to April 2016. The study protocol was approved by Pasteur Institute of Iran Ethical Committee, and an informed consent was obtained from each subject prior to the enrollment. Eligibility criteria included HIV infection, initial CD4 count of 350 cells/mm^3^, and baseline age of at least 15 years. However, individuals with AIDS-defining illnesses and patients receiving antiretroviral therapy, anti-HSV treatment, and immunomodulatory drugs were excluded. Study participants and studying steps are shown in Figure 1.

### Flow cytometry

CD4 count was determined by flowcytometry using BD FACSCalibur (BD Biosciences Company, USA) and defined as cells/mm^3^.

### Real-time assay

Serum HIV-RNA copy numbers were determined by real-time PCR using RealStar HIV RT-PCR Kit (altona Diagnostics GmbH, Hamburg, Germany) on the Rotor-Gene 6000 real-time thermal cycler (Corbett Research, Sydney, Australia).

### Detection of HSV-2 antibodies

All plasma samples were tested for HSV-2 IgG-specific antibody using ELISA (EUROIMMUN, Lübek, Germany). All assays were performed according to the protocols provided by manufacturer.

### Statistical analysis

Statistical analyses were conducted using SPSS statistics software (version 16, Chicago, IL, USA). The Chi-square test or Mann-Whitney U test was used to compare variables. Data were presented as mean±SD or when indicated, as an absolute number and percentage. Mixed linear regression models were used to assess the effect of HSV-2 on log HIV viral load and CD4 count. *p* values <0.05 were considered statistically significant.

## RESULTS

A total of 116 naïve HIV-infected patients with the mean age of 34.33 ± 8.2 years and 85 healthy controls with the mean age of 35.9 ± 5.67 years were enrolled in the study. In HIV positive group, the baseline mean CD4 count and HIV viral load were 664.73 ± 254.94 cells/mm^3^ and 4.90 ± 5.44 log copies/mL, respectively. The reported routes of HIV transmission were intravenous drug use (38.3%), heterosexual contact (60.9%), infected blood and blood products (1.7%), vertical transmission (0.9%), homosexual (1%), tattoo (22.6%), and in 12.2%, the route of HIV acquisition was not identified. The prevalence of HSV-2 IgG was 18.1% in HIV cases and 0% in the control group (*p* = 0.000). There was no significant difference regarding baseline CD4 count between HSV-2 seronegative and seropositive groups (670.40 ± 263.95 vs. 639.38 ± 213.77 cells/mm^3^, respectively; *p* = 0.76). The mean baseline HIV viral load was 4.48 ± 4.73 log copies/mL in HSV-2 seropositive and 4.96 ± 6.48 log copies/mL among HSV-2 seronegative cases without any significant difference (*p* = 0.57). HSV-2 seroconversion was observed in 2.43% of HIV patients after one year.

Rates of CD4 count and HIV viral loads changing were compared with HSV-2 status using mixed linear regression models. There was not any statistically significant association between HSV-2 serostatus and CD4 count over time (*p* = 0.85). Moreover, HSV-2 co-infection was not associated with HIV viral loads changing in naïve HIV patients after one year follow-up (*p* = 0.23). Comparison of HSV-2 seropositive and seronegative HIV-infected patients at baseline and after one-year follow-up is shown in Table 1.

**Table 1:** Comparison of HSV-2 seropositive and seronegative HIV-infected patients at baseline and after one-year follow-up

HIV-infected patients	HSV-2 seropositive	HSV-2 seronegative	*p* value
CD4 at baseline (cells/mm^3^)	639.38 ± 213.77	670.40 ± 263.95	0.76
Viral load at baseline ( log copies/mL)	4.48 ± 4.73	4.96 ± 6.48	0.57
CD4 after one-year follow-up (cells/mm3)	550.56 ± 248.05	563.48 ± 228.43	0.74
Viral load after one-year follow-up (log copies/mL)	3.97 ± 1.35	3.49 ± 1.72	0.74

HSV, Herpes simplex virus type

## DISCUSSION

In this study, we investigated the seroprevalence of HSV-2 IgG in naïve HIV patients and healthy control group in Tehran, Iran. We also evaluated the effect of HSV-2 seropositivity on HIV viral load and CD4 count at baseline and after one-year follow-up. The prevalence of HSV-2 IgG was 18.1% in naïve HIV patients and 0% in the control group. HSV-2 seroconversion was observed in 2.43% of HIV patients after one year. Moreover, we showed that co-infection with HSV-2 had no association with CD4 count and HIV RNA viral load changing in our study cohort at baseline or over time.

HSV-2 infection is the most common genital ulcer disease in HIV patients. High seroprevalence of HSV-2 in HIV individuals has been reported from different parts of the world [24], 63-77% in the USA[24,25], 81% in the UK[26], 73% in Brazil[27], and 88% in China[28]. The present study showed the low prevalence of HSV-2 infection in HIV subjects (18.1%) and normal controls (0%). However, there are limited data on the prevalence and incidence of HSV-2 in Iranian HIV patients. A few studies were conducted in some high-risk and low-risk groups in Iran, which is in agreement with our results. Asgari *et al*.[29] reported HSV-2 IgG among women attending obstetrics and gynecology clinics and prisoners’ women as 26.3% and 2.5%, respectively. Another survey carried out by Navadeh *et* *al*.[30] on female sex workers in southeast of Iran showed the rate of 18% for HSV-2 prevalence.

Few data exist about HSV-2 seroconversion in HIV patients. A survey regarding HSV-2 seroconversion in HIV patients revealed an HSV-2 seroincidence of 1.8 per 100 person-years[31]. Cachay *et al*.[32] determined HSV-2 seroconversion in 119 HAART naïve HIV-infected men in a retrospective study and showed that 8.4% of the cases acquired HSV-2 infection with a median of 1.3 years after HIV infection (HSV-2 incidence rate of 7.4 per 100 person-years of follow-up). In a study in Brazil on high-risk groups for sexually transmitted diseases, HSV-2 seroprevalence and annual incidence rates were 67% and 0.08%, respectively[33]. The present study also found HSV-2 seroconversion in only 2.43% of HIV patients after one-year follow-up, which is in agreement with Ramaswamy *et al*. study[31].

Both HIV and HSV-2 viruses interact to each other. HIV unfavorably can change the natural course of HSV-2 infection and lead to more frequent and severe HSV-2 reactivation[18]. Although HSV-2 infection has infrequent sequelae and complications, numerous epidemiological studies have shown that it facilitates HIV acquisition and transmission by threefold, and few studies have described the possible effects of HSV-2 infection on HIV progression[19,34,35]. Duffus *et al*. [17] found that HSV-2/HIV co-infection was significantly associated with higher HIV viral load in comparison to individuals without HSV-2 infection. Therefore, HSV-2 infection may have a negative role on the clinical course of HIV patients. Besides, some investigations on homosexual men have demonstrated the raising levels of HIV RNA in lesions after HSV-2 ulcerations[36] and transient increasing effect on plasma HIV RNA levels during clinical or subclinical HSV-2 reactivations[36,37]. Nagot *et al*.[19] findings suggested that both clinical and subclinical HSV-2 reactivations have effect on increasing HIV replication. Additionally, a dose-dependent association between the markers of HIV disease progression and the degree of HSV-2 clinical activity was described in Aumakhan study[38]. In contrast, Hoots *et al*.[18] suggested that HSV-2 did not notably influence on the changing the HIV viral load and CD4 count over time among HSV-2 seropositive and seronegative cases who were not treated with HIV. Similar results were found in an investigation in California[22] and another study in Uganda[16]; both observed no difference between HIV viral load and HSV-2 serostatus in prevalent HIV infection. Tan *et al*.[39] also reported that HSV-2/HIV co-infection has no role in decreasing CD4 count in naïve HIV patients. Ramaswamy *et al*.[31] evaluated the HSV-2 seroconversion rate and found HSV-2 acquisition could not significantly influence the plasma HIV viral load and CD4 count after one-year follow-up.

Our findings are in agreement with previous studies[16,18,22,31,39]. However, definitive evidence and conclusive relationship regarding HSV-2 seropositivity and accelerated HIV disease progression are still lacking. In conclusion, our findings indicated that the prevalence and incidence of HSV-2 infection is low in our HIV naïve patients relative to the patients from other countries, and HSV2 situation is negligible in the control group. Moreover, it does not seem that HIV/HSV2 co-infection plays a role on HIV infection progression.
